# Long Non-Coding RNAs in Hepatitis B Virus-Related Hepatocellular Carcinoma: Regulation, Functions, and Underlying Mechanisms

**DOI:** 10.3390/ijms18122505

**Published:** 2017-11-23

**Authors:** Lipeng Qiu, Tao Wang, Xiuquan Xu, Yihang Wu, Qi Tang, Keping Chen

**Affiliations:** 1 Institute of Life Sciences, Jiangsu University, Zhenjiang 212013, China; theowang18@163.com (T.W.); tangqi1224@163.com (Q.T.); 2 School of Pharmacy, Jiangsu University, Zhenjiang 212013, China; xxq781026@ujs.edu.cn; 3 Department of Pharmacy, College of Life Sciences, China Jiliang University, Hangzhou 310018, China; yihangwu@126.com

**Keywords:** lncRNAs, HBV, HBx, HCC, proliferation, epithelial-mesenchymal transition, invasion and metastasis, apoptosis, autophagy

## Abstract

Hepatocellular carcinoma (HCC) is the fifth most common cancer and the third leading cause of cancer death in the world. Hepatitis B virus (HBV) and its X gene-encoded protein (HBx) play important roles in the progression of HCC. Although long non-coding RNAs (lncRNAs) cannot encode proteins, growing evidence indicates that they play essential roles in HCC progression, and contribute to cell proliferation, invasion and metastasis, autophagy, and apoptosis by targeting a large number of pivotal protein-coding genes, miRNAs, and signaling pathways. In this review, we briefly outline recent findings of differentially expressed lncRNAs in HBV-related HCC, with particular focus on several key lncRNAs, and discuss their regulation by HBV/HBx, their functions, and their underlying molecular mechanisms in the progression of HCC.

## 1. Introduction

Hepatocellular carcinoma (HCC) is the fifth most common cancer and the third leading cause of cancer death in the world, and represents over 90% of primary liver cancers. The leading cause of HCC is persistent hepatitis B virus (HBV) infection, which happen in more than half of HCCs [1,2]. Once this occurs, the HBV infection may become chronic, and HBV genome may integrate into the host genome, which are supposed to promote initiation and progression of HCC [3,4]. Among the four open reading frames of the HBV genome, the smallest one, HBX, is translated into a protein named hepatitis B virus X protein (HBx). It has been found to contribute to the pathogenesis of HCC by trans-modulating many growth regulatory genes and activating various signaling pathways, including p53, NF-κB, and Wnt signalling [5,6].

Non-coding RNAs (ncRNAs), which represent the majority of human genome transcripts, were originally believed to be “junk RNAs” because they cannot code proteins. According to the length, ncRNAs are classified into two groups: small ncRNAs (18–200nt), such as microRNAs (miRNAs) and piwiRNAs (piRNAs), and long ncRNAs (lncRNAs, 200–100kb). miRNAs, which regulate gene expression by impacting target messenger RNA (mRNA) degradation or protein translation, have been found to be dysregulated and play important roles in HBV infection and HCC progression [7]. For example, miR-122 was found to be frequently downregulated in HBV-related HCC and HBV-expressing hepatic cells, and could not only suppress HBV replication, but also inhibit different aspects of HCC progression by modulating the expression of various target genes, such as cyclin G1, ADAM10, IGF1R, SRF, HMOX1, and PTTG1. [8]. Another miRNA, miR-125a, may be upregulated by HBV through HBx, whereas over-expression of miR-125a, in turn, inhibits HBV replication by directly targeting viral transcripts [9]. Intriguingly, increasing evidence has indicated that lncRNAs also play essential roles in various biological processes, including cell proliferation, invasion and metastasis, autophagy, and apoptosis, and thus contributes to the progression of different human cancers, including HCC [10]. However, the functions and mechanisms of lncRNAs in HBV-related HCC remain to be fully elucidated. 

**Figure 1:**
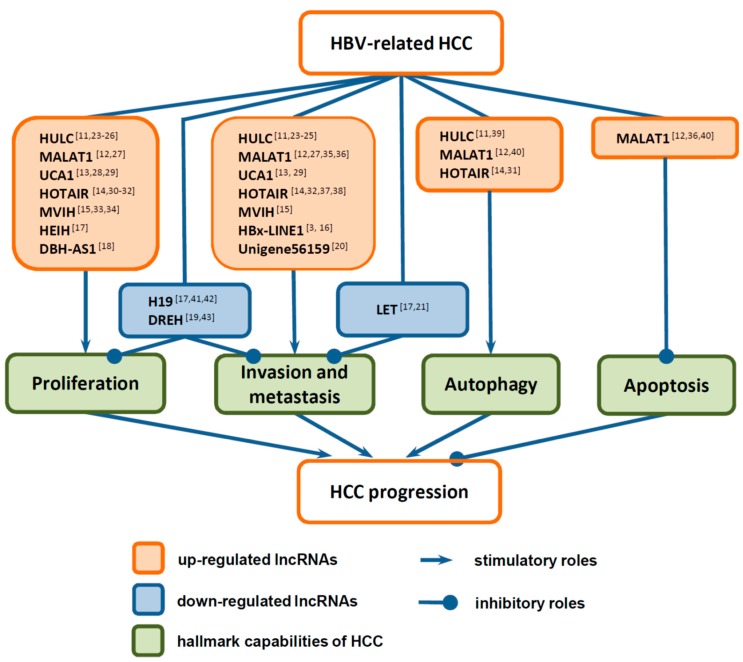
Dysregulated long non-coding RNAs (lncRNAs) in hepatitis B virus (HBV)-related hepatocellular carcinoma (HCC) and their functions in HCC progression, including the modulation of HCC cell proliferation, invasion and metastasis, autophagy, and apoptosis [3,11,12,13,14,15,16,17,18,19,20,21,23,24,25,26,27,28,29,30,31,32,33,34,35,36,37,38,39,40,41,42,43].

Most recently, several lncRNAs were reported to be frequently dysregulated in HBV-related HCC, including HOTAIR (HOX transcript antisense intergenic RNA), MALAT1 (metastasis-associated lung adenocarcinoma transcript 1), H19 (imprinted maternally expressed untranslated mRNA), UCA1 (urothelial carcinoma associated-1), HULC (highly upregulated in liver cancer), MVIH (microvascular invasion in HCC), HBx-LINE1 (fusion of the human cellular long interspersed nuclear elements and HBx), HEIH (highly expressed in HCC), DBH-AS1 (DBH antisense RNA 1), DREH (downregulated by HBx), unigene56159, and LET (low expression in tumor) [11,12,13,14,15,16,17,18,19,20,21]. Some of these lncRNAs, including HOTAIR, MALAT1, H19, UCA1, HULC, and MVIH, have been demonstrated to be dysregulated in various cancers, whereas others have only been found to be dysregulated in HCC [10,22]. Functional studies revealed that these lncRNAs contribute to the onset and progression of HBV-related HCC (Figure 1). In this review, we briefly outline the recent findings of these differentially expressed lncRNAs in HBV-related HCC, with particular focus on several key lncRNAs, and discuss their regulation by HBV/HBx, their functions, and their underlying molecular mechanisms in HCC progression.

## 2. Dysregulation of LncRNAs in HBV-Related HCC and Their Regulation by HBV/HBx

Among all the listed lncRNAs, many were frequently dysregulated in both HBV-related HCC tissues and HBV-/HBx-expressing cell lines, while a few were identified to be dysregulated in HBV-related tissues. For example, HULC, MALAT1, UCA1, and DBH-AS1 were upregulated in HBV-related HCC tissues and HBx-expressing hepatic cell lines, and their expression as positively correlated with those of HBx in HBV-related HCC tissues [11,12,13,18]; HOTAIR was also increased in HBV-related HCC and live tumors of X/c-myc transgenic mice [14]. Unigene56159 was found to be elevated in HBV-related HCC and HBV-producing cell line [20], whereas MVIH and HEIH were detected to be upregulated in HBV-related HCC tissues [15,17]. A special transcript, HBx-LINE1, a consequence of the integration of HBV into the human genome, is fused by the human LINE (1) and HBx [16]. It could be detected in 23.3% of HBV-related HCC patients [16]. Three frequently downregulated lncRNAs in HBV-related HCC include DREH, H19, and LET. Among them, DREH was downregulated in HBV-related HCC, HBx-transgenic mice, and HBx-expressing mouse liver cells; LET was identified as being decreased in HBV-related HCC, whereas H19 was controversially reported to be decreased in HBV-related HCC while upregulated in HCC [17,19,21,41].

While most studies merely mention the dysregulation of lncRNAs by HBV or HBx, only two have discussed the underlying mechanisms. One is about HOTAIR, and the other is about HULC. The transcription regulation of HOTAIR by HBV/HBx are associated with the modulation of two transcription repressive complexes, polycomb repressive complex 2 (PRC2), and LSD1/Co-REST/HDAC1, which are held together by binding HOTAIR [14]. In HBV-replicating cells, the mitotic polo-like kinase 1 (Plk1) was found to be activated by HBx, and the activation of Plk1 induces proteasomal degradation of SUZ12, an essential subunit of PRC2 complex, and ZNF198, a protein which is capable of stabilizing the LSD1/Co-REST/HDAC1 complex, resulting in chromatin modification and leading to the activation of HOTAIR transcription [14]. The transcription regulation of another lncRNA, HULC, by HBV/HBx, was mediated by the transcription factor cAMP responsive element binding protein (CREB) [11,44]. CREB has been reported to recruit P300 and Brg into the *HULC* promoter, leading to the activation of epigenetic markers and chromatin remodeling and resulting in the transcription of HULC [44]. By interacting with CREB, HBx activates the *HULC* promoter activity and thus increases HULC transcription [11]. Meanwhile, enhanced HULC also activates CREB by inhibiting miR-372 and increasing the expression of the miR-372 target gene cAMP-dependent protein kinase catalytic subunit beta (PRKACB), which, in turn, enhances the phosphorylation of CREB and, consequently, HULC transcription [44].

## 3. Functions of LncRNAs in HCC Progression

### 3.1. Functions of LncRNAs in the Proliferation of HCC Cells

Among the listed lncRNAs, a major part has been shown to modulate the proliferation of HCC cells in vitro and in vivo. In in vitro studies, the alteration of lncRNA expression by over-expression or knockdown may result in altered proliferative capacities and colony formation abilities of HCC cells. For example, over-expression of lncRNAs UCA1 or DBH-AS1 significantly increased, while knock-down of these lncRNAs greatly inhibited, the proliferation and colony formation of HCC cells [13,18,28,29]; enhanced expression of HULC or HOTAIR promoted, while silencing of these lncRNAs reduced, the proliferation of HCC cells [11,23,24,30,31,32]; siRNA or shRNA against H19, MVIH, HEIH, and MALAT1 successfully inhibited the viability of HCC cells [17,27,33,41], whereas siRNA against DREH, however, enhanced the proliferation effect and colony formation ability of HCC cells [19,43]. In in vivo studies, HCC cells with the altered expression of some lncRNAs were injected into nude mice for xenotransplantation, and the corresponding tumor volume, size, and weight were found to be altered when compared with those of tumors formed from control xenografts. For instance, HCC cells over-expressing lncRNAs HEIH, HOTAIR, MVIH, HULC, or DBH-AS1 resulted in promoted tumor growth, while HCC cells depleting these lncRNAs resulted in markedly suppressed tumor growth [11,15,17,18,24,25,30,33]; silencing of lncRNAs H19 or UCA1 led to reduced tumor growth [13,28,41], whereas DREH over-expressing HCC cells decreased, while DREH knocking-down HCC cells enhanced, tumor growth when compared with those of tumors formed from control xenografts [43]. These results suggested that HCC cells proliferation and tumor growth could be modulated by over-expressing the tumor suppressive gene DREH, or inhibiting the expression of oncogenes, such as HULC and HOTAIR.

### 3.2. Functions of LncRNAs in Invasion and Metastasis of HCC Cells

Most of the listed lncRNAs in this review have been suggested to regulate migration, invasion, and metastasis of HCC cells. In vitro studies, transwell migration assays, wound-healing assays, and matrigel invasion assays were used to evaluate the effects of lncRNAs on the migration and invasion of HCC cells, while a human umbilical vein endothelial cell (HUVEC) tube formation assay was used to evaluate the effects on tumor angiogenesis. Over-expression of several lncRNAs, including MALAT1, HOTAIR, H19, and unigene56159, have been demonstrated to increase, while the knocking down of these lncRNAs has been shown to decrease the migration and invasion capacity of HCC cells [12,20,27,32,35,37,42]; the promotion of UCA1 greatly enhanced, while inhibition of this lncRNA significantly reduced, the invasion ability of HCC cells [29], whereas the silencing of HULC or HBx-LINE1 significantly reduced the migration and invasion activity of HCC cells [16,24]. However, over-expression of LET inhibited, while depletion of this lncRNA or DREH facilitated, the invasion capacity of HCC cells [19,21]. In addition, upregulated HULC or MVIH was capable of activating HCC cells to promote the tube formation of HUVECs [15,25]. In in vivo studies, several mice models, including intrahepatic, orthotopic implanted, and peripheral intravascular implanted metastatic models, were used to evaluate the effects of lncRNAs on the invasion and metastasis of HCC cells. In the intrahepatic metastatic model, HCC cells with altered lncRNAs were subcutaneously injected into livers of nude mice; in the orthotopically implanted metastatic model, subcutaneous tumor tissues derived from HCC cells with altered lncRNAs were used for intrahepatic transplantation in the nude mice, whereas, in the peripheral intravascular implanted metastatic model, HCC cells with altered lncRNAs were injected into nude mice through the tail vein. Three lncRNAs, including DREH, LET, and H19, exhibited a significant reduction on invasion and metastasis of HCC cells in vivo [19,21,42]. LET over-expressing HCC cells resulted in dramatically decreased, while LET knocking-down HCC cells displayed increased, intrahepatic metastasis and pulmonary metastasis in orthotopic and peripheral intravascular implanted metastatic models [19]. Similarly, DREH over-expressing HCC cells demonstrated significant reduced intrahepatic metastasis and less definite pulmonary metastasis sites in these models [21]. HCC cells also depleted H19, facilitated intrahepatic metastasis, and manifested significant cachexia and weight loss in orthotopic implanted metastatic mice [42]. MVIH and HULC remarkably increased invasion and metastasis of HCC cells in vivo [15,24]. MVIH over-expressing HCC cells frequently led to enhanced intrahepatic metastasis in intrahepatic and orthotopic implanted metastatic models [15], whereas HULC knocking-down HCC cells produced less intrahepatic metastasis in intrahepatic metastatic models [24]. These data indicated that the invasive and metastatic properties of HCC cells could be blocked by over-expressing tumor-suppressive genes, such as DREH or LET, or by inhibiting the expression of oncogenes, such as HULC and MVIH.

### 3.3. Functions of LncRNAs in Death of HCC Cells

Both autophagy and apoptosis are recognized as programmed cell death (PCD). Autophagy is a protein degradation process that begins with the formation of autophagosomes and is followed by their fusion with lysosomes to form autolysosomes to dispose of their contents [45]. It could be detected by directly observing autophagosomes via transmission electron microscopy (TEM), indirectly testing the expression their surface marker microtubule-associated protein 1 light chain 3 (LC3)/fluorescence-labelled LC3, or indirectly detecting the expression of autophagy cargo receptor and substrate sequestasome 1 (SQSTM1, p62). Several lncRNAs have been suggested to modulate autophagy of HCC cells. For example, over-expression of HOTAIR or HULC increases, while silencing of HOTAIR or MALAT1 reduces, autophagy of HCC cells [31,39,40]. Unlike autophagy, during the process of apoptosis, caspases are activated, which then kill the cell by degrading proteins indiscriminately. Thus, Annexin V/PI double staining, caspase-3 activity, or terminal transferase-mediated dUTP fluorescein nick end-labelling (TUNEL) assays may be used to determine the apoptosis of HCC cells. LncRNA MALAT1 was reported to inhibit apoptosis of HCC cells [40]. Additionally, chemosensitivity of HCC cells also impact the survival of HCC cells. For example, upregulation of HULC in HCC cells decreases their sensitivity to oxaliplatin and facilitates their survival, whereas the knocking down of MALAT1 in chemoresistant HCC cells promotes 5-fluomuraeil-induced apoptosis of these cells and reduces their survival [39,40]. These results demonstrated that the survival of HCC cells could be suppressed by increasing their apoptosis and chemosensitivity or by inhibiting their autophagy. 

## 4. Underlying Mechanisms of LncRNAs in HCC Progression

### 4.1. Underlying Mechanisms of LncRNAs in the Proliferation of HCC Cells

In the tumorigenesis of HCC, the dysregulation of cell cycle control is an essential step, which leads to an uncontrolled cell proliferation. Cell cycle consists of four phases: G1, S (synthesis), G2, and M (mitosis). The progression of cell cycle is governed by cyclin-dependent kinases (CDKs) and their cyclin partners, which form cyclin/CDK complexes to perform their functions. In these complexes, cyclins form the regulatory subunits, which are changed to aid the transition between cycle phases, whereas CDKs form the catalytic subunits, which can be bound and activated by cyclins to facilitate cell cycle progression. Activated CDKs phosphorylate specific substrates and initiate the transition from the G1 phase to the S phase and from the phase G2 to the M phase, thus promoting cell cycle progression. In contrast, two families of genes, including inhibitors of CDK4 (INK4) family (p15^INK4b^, p16^INK4a^, p18^INK4c^,p19^INK4d^) and CDK-interacting protein/kinase inhibition protein (Cip/Kip) family (p21^CIP^, p27^KIP1^, p57^KIP2^), prevent the progression of cell cycle by binding and inactivating cyclin/CDK complexes. Consequently, different molecules, such as proteins and non-coding RNAs, may participate in cell cycle control by modulating the expression of cyclins, CDKs, and CDK inhibitors (CDKIs) and thus impact on the proliferation of HCC cells.

Recently, several dysregulated lncRNAs in HBV-related HCC have been demonstrated to regulate the cell cycle by directly or indirectly modulating the expression of cyclins, CDKs, CDKIs, and thus contribute to the proliferation of HCC cells. For example, knocking-down of H19 facilitates cell proliferation by decreasing the CDKI p57 and abolishing its induction in response to hypoxic stress; MVIH promotes cell proliferation by upregulating the expression of Frizzled type 7 receptor (FZD7), its downstream genes CCND1 (a member of cyclins), and Myc through sponging miR-199a [33,34,41]. Similarly, HULC increases HCC cell proliferation by inhibiting CDKI p18 and increasing CCND1 and CCNE1 (member of cyclins) [11,23,26]. By directly binding to the promoter region of p18, HULC inhibits its promoter activity and protein expression; by specifically binding to and promoting the phosphorylation of Y-box binding protein (YB-1), HULC releases CCND1 and CCNE1 mRNAs, resulting in their translation activation [11,23,26]. Three lncRNAs, HEIH, UCA1, and HOTAIR, were reported to promote HCC cell proliferation by repressing CDKIs through association with enhancer of zeste homolog 2 (EZH2), a component of PRC2 [13,17,30]. By associating with EZH2, HEIH represses CDKIs p16 and p21, and thus plays a key role in G0/G1 arrest and promotes tumor progression; UCA1 inhibits CDKI p27, leading to increased expression of CDK2 (a member of CDKs), thus facilitating G1/S transition and accelerating cell cycle progression, whereas HOTAIR decreases p16 and p14 through inhibiting miR-218 expression and increasing Bmi-1 expression and thus contributes to hepatocarcinogenesis [13,17,30]. Furthermore, lncRNAs, including UCA1, DBH-AS1, and MALAT1, also facilitate proliferation of HCC cells by mediating cell cycle progression through modulating different signaling pathways, such as ERK and PI3K/Akt pathways [18,27,28]. UCA1 promotes the ERK signaling pathway by sponging miR-216 and increasing the expression of miR-216 target gene fibroblast growth factor receptor 1 (FGFR1); DBH-AS1 activates the ERK/p38/JNK MAPK signaling pathway, inducing the expression of CDK6 (a member of CDKs), CCND1, and CCNE1 and reducing the expression of p16, p21, and p27; whereas MALAT1 accelerates the PI3K/Akt pathway by sponging miR-195 and increasing the expression of miR-195 target gene epidermal growth factor receptor (EGFR) [18,27,28].

### 4.2. Underlying Mechanisms of LncRNAs in Invasion and Metastasis of HCC Cells

Metastasis, the main reason for poor prognosis and survival rates of HCC patients, is formed by a complicated succession of invasion–metastasis cascades. The first step of this cascade is typically believed to be the epithelial–mesenchymal transition (EMT), a biological process involving a functional transition of polarized epithelial cells into the mobile and extracellular matrix component-secreting mesenchymal cells. During EMT, epithelial cells lose their cell–cell contacts, undergo a switch of marker protein expression from epithelial (E-cadherin, cytokeratin, and γ-catenin) to mesenchymal (*N*-cadherin, vimentin, and fibronectin) cells, and gain increased migratory and invasive capabilities [46]. These cells may further spread into surrounding or distant tissues, anchor themselves, re-gain an epithelial-like state, and then form macrometastases. A group of transcription factors, including snail family zinc finger (Snail1 and Snail2), zinc finger E-box binding homeobox (ZEB1 and ZEB2), and twist basic helix-loop-helix transcription factor (Twist), has been shown to induce EMT [47,48]. Signaling pathways, such as TGF-β and Wnt/β-catenin pathway, also contribute to EMT and subsequently metastasis [49,50]. Moreover, the tumor microenvironment, including surrounding blood vessels, cytokines, and the extracellular matrix proteins, such as matrix metalloproteinase (MMP), also influence the metastasis of HCC cells [51].

Several dysregulated lncRNAs in HBV-related HCC have been found to regulate invasion and metastasis of HCC cells by modulating EMT-related genes, different signaling pathways, and tumor microenvironments. For example, DREH, unigene56159, UCA1, H19, and HBx-LINE1 contributes to invasion and metastasis by modulating EMT. Specifically, DREH directly targets mesenchymal marker vimentin and suppresses its expression, leading to the inhibition of tumor cell migration and invasion; unigene56159 and UCA1 enhance the expression of EMT-associated transcription factor Snail2 by targeting and sequestering miR-140-5p and miR-203, respectively, leading to decreased expression of E-cadherin and cytokeratin, as well as increased expression of vimentin and, thus, facilitating EMT and invasion; H19 inhibits the expression of EMT-associated transcription factor ZEB1/2 by increasing the expression of miR-200 family through facilitating epigenetic modulation, whereas HBx-LINE1 depletes miR-122, promotes EMT-like changes, including the activation of Wnt/β-catenin signaling, E-cadherin suppression, cell migration enhancement, and thus promotes the invasion and metastasis of HCC cells [3,16,19,20,29,42]. Furthermore, MVIH and HOTAIR facilitate invasion and metastasis by modulating the tumor microenvironment. MVIH suppresses the secretion of phosphoglycerate kinase 1 (PGK1), a protein inhibiting angiogenesis, leading to activation of angiogenesis, which promotes tumor growth and intrahepatic metastasis, whereas the function of HOTAIR on the migration and invasion of HCC cells is associated with decreasing the expression of MMP-9 and vascular endothelial growth factor (VEGF), as well as increasing the expression of RNA binding motif protein 38 (RBM38) [15,37,38]. Additionally, lncRNAs also function by modulating other proteins associated with invasion and metastasis. For example, LET is associated with nuclear factor 90 (NF90), which regulates many factors related to tumor growth and metastasis, leading to reduced expression of NF90 and its downstream target gene hypoxia-induced factor 1α, and resulting in inhibition of hypoxia-induced invasion of HCC cells [21]. Moreover, both HULC and MALAT1 facilitate the invasion and metastasis of HCC cells through multiple mechanisms. HULC promotes invasion and metastasis by targeting YB-1 and miR-200a-3p, leading to increased expression of MMP-3 and transcription factor ZEB1, respectively; it also sponges miR-107 and enhances the expression of its target gene E2F1, leading to increased expression of sphingosine kinase 1 (SPHK1) and sphingosine-1-phsphate (S1P), which result in enhanced tumor angiogenesis [23,24,25]. Another lncRNA, MALAT1, also facilitates tumor growth and metastasis by modulating multiple proteins, miRNAs, and signaling pathways [12,27,35,36,52,53]. Firstly, MALAT1 sponges miR-204 and releases its suppression on silent information regulator 2 homolog 1 (SIRT1), which, in turn, regulates EMT and enhances the invasion and metastasis of HCC cells [35,52]. Secondly, MALAT1 sequesters miR-146b-5p, leading to upregulated tumor necrosis factor (TNF) receptor associated factor 6 (TRAF6) and TRAF6-mediated AKT phosphorylation, resulting in enhanced expression of MMP-9, which facilitates the migration of HCC cells [36]. Thirdly, MALAT1 sponges miR-195 and increases the expression of EGFR, resulting in the activation of PI3K/AKT and JAK/STAT pathways [27]. Lastly, MALAT1 may increase the expression of latent transforming growth factor (TGF) β-binding protein (LTBP) 3, a protein which could bind to TGF-β and is associated with TGF-β extracellular matrix secretion, deposition, and latency [12,53].

### 4.3. Underlying Mechanisms of LncRNAs in Death of HCC Cells

Autophagy and apoptosis play major roles in determining cell fate. Autophagy is a highly conserved cytoprotective process in response to various cellular stresses, such as starvation, hypoxia, and chemotherapy, whereby cytoplasmic content self-degrades and proper cell function and survival is maintained [54,55]. Autophagy-related genes (ATGs), for example, ATG3 and ATG7, play essential roles in autophagy [56]. Other factors, such as SIRT1 and miR-495, were found to regulate ATGs and thus contribute to autophagy [57,58,59]. By contrast, apoptosis is a highly regulated cell-death process that can be initiated when it senses intercellular stress, such as oxidative stress, DNA damage, hypoxia, and growth-factor deprivation, or extrinsic signals that could activate cell-surface death receptors, such as specific ligands, to kill the cell by degrading proteins indiscriminately. Factors such as Fas receptors and caspases increase apoptosis, whereas some members from the B-cell lymphoma 2 (Bcl-2) family inhibit this process. In spite of the great differences between autophagy and apoptosis, they are intimately connected to each other and share intersecting protein networks. Under the microenvironment of HCC, unbalanced autophagy and apoptosis contribute to carcinoma cell survival, impacting on the sensitivity of chemotherapy reagents and, in turn, have an effect on tumor growth and development [60,61,62].

LncRNAs, including HOTAIR, HULC, H19, and MALAT1, were reported to promote HCC progression by activating autophagy, reducing apoptosis, or reducing the chemosensitivity of HCC cells. HOTAIR promotes the activation of autophagy by increasing the expression of ATG3 and ATG7, sensitizes TNF-α induced apoptosis, and reduces the sensitivity of the HCC cells to chemotherapeutic reagents, such as cisplatin and doxorubicin; HULC could be greatly upregulated by antitumor reagents such as oxaliplatin, 5-fluorouracil, and pirarubicin, and then trigger protective autophagy and weaken the chemosensitivity of HCC cells by targeting a group of miRNAs, including miR-6825-5p, miR-6845-5p, and miR-6886-3p, resulting in over-expression of ubiquitin-specific peptidase 22 (USP22) and increased stability of SIRT1 [31,32,39]. H19 reduces the chemosensitivity of HCC cells to doxorubicin by regulating the methylation of the multi-drug resistance 1 (MDR1) promoter and induces its expression, whereas lncRNA MALAT1 could be upregulated in multi-drug resistance (MDR) HCC cells and could then promote autophagy, inhibit apoptosis, and reduce chemosensitivity by sequestering miR-216 and miR-146b-5p and subsequently by activating TRAF6/p-Akt signaling, increasing the expression of anti-apoptotic protein Bcl2 and myeloid cell leukemia-1 (Mcl-1) [36,40,63].

## 5. Conclusions and Future Perspectives

**Figure 2:**
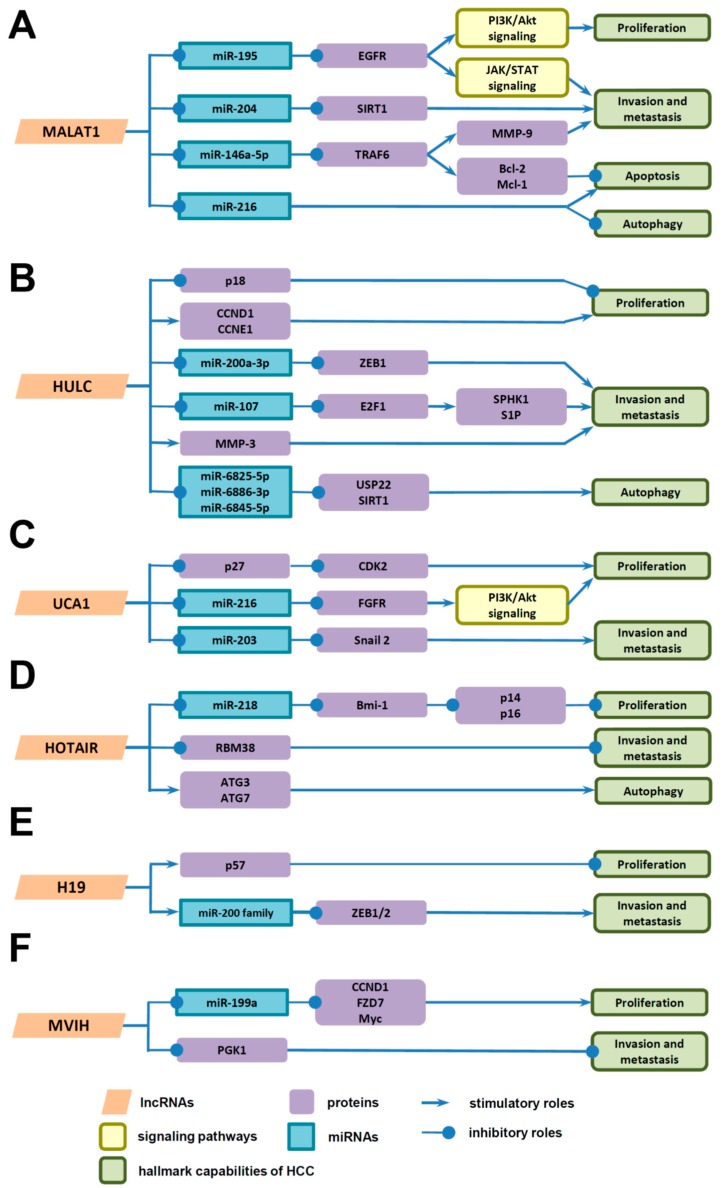
Several key lncRNAs aberrantly expressed in HBV-related HCC, their targets, and their underlying molecular mechanisms in HCC progression. (**A**) MALAT1 (metastasis-associated lung adenocarcinoma transcript 1); (**B**) HULC (highly upregulated in liver cancer); (**C**) UCA1 (urothelial carcinoma associated-1); (**D**) HOTAIR (HOX transcript antisense intergenic RNA); (**E**) H19 (imprinted maternally expressed untranslated mRNA); (**F**) MVIH (microvascular invasion in HCC).

Growing evidence indicates that some lncRNAs are dysregulated in HBV-related HCC and HBV-/HBx-expressing hepatic cells, and their dysregulation is correlated with clinicopathological features in HCC patients. By functioning as signal, decoy, guide, or scaffold, these lncRNAs regulate gene expression at epigenetic, transcriptional, and post-transcriptional levels and mediate signaling, such as phosphorylation and trafficking of proteins, thus contributing to diverse biological processes involved in HCC progression, including proliferation, invasion and metastasis, autophagy, and apoptosis [64]. In this review, we summarized recent findings of these dysregulated lncRNAs in HBV-related HCC, their regulation by HBV/HBx, and their functions and mechanisms of action in the progression of HCC. The specific targets and mechanisms of action of several key lncRNAs are shown in Figure 2.

When more lncRNAs were found to be dysregulated in HCC, only part of them have been identified to be dysregulated in HBV-related HCC [10]. Among these identified lncRNAs, although many have been demonstrated to be modulated by HBV/HBx, their underlying regulatory mechanisms are still unclear. Current research suggests that lncRNAs play important roles in HBV/HBx-induced pathogenesis of HCC. Some lncRNAs, such as HULC and HOTAIR, could be upregulated by HBV/HBx, and the upregulation of these lncRNAs promotes HCC progression by accelerating cell proliferation, invasion and metastasis, and autophagy and by reducing apoptosis and chemosensitivity through regulating the expression of multiple protein-coding genes, miRNAs, as well as different signaling pathways. A unique example is HBx-LINE1, a particular chimeric transcript of virus DNA and host sequence LINE1, which gives evidence that HBV DNA may impact the progression of HBV-related HCC by integrating into the genomes of host cells. Furthermore, lncRNAs may play a role in HBV replication and host anti-HBV responses of IFN. For example, HBx-LINE1 suppresses miR-122, a miRNA that has been demonstrated to inhibit HBV replication by directly targeting the HBV pregenomic RNA sequence or by indirectly modulating HO-1 and CCNG1/p53 pathways [65,66,67]. Additionally, since miR-122 could be inhibited by interferon-α, it also negatively affects anti-HBV efficiency of interferon-α [68]. It is thus possible that, by depleting miR-122, HBx-LINE1 promotes HBV replication but plays a negative role in the anti-HBV response of interferon-α. Another lncRNA, lncRNA#32, could positively regulate IFN-stimulated gene expression by interacting with activating transcription factor 2 (ATF2) and thus significantly inhibit HBV replication [69]. Furthermore, it remains to be clarified whether HBV genotypes (10 genotypes, A–J) influence the dysregulation of lncRNA [70]. Given the important roles of lncRNAs in HBV-induced progression of HCC, more extensive studies are required to develop a precise profile of HBV/HBx regulated lncRNAs. Furthermore, the regulatory mechanisms of these lncRNA by HBV/HBx, their linkages with HBV replication, host immune responses, and the aetiology of HCC, as well as their function and underlying molecular mechanisms in HCC progression need to be fully elucidated.

## Abbreviations

HCC	hepatocellular carcinoma
HBV	hepatitis B virus
HBx	hepatitis B virus X protein
ncRNA	non-coding RNA
miRNA	microRNA
lncRNA	long non-coding RNA
CDK	cyclin-dependent kinase
CDKI	cyclin-dependent kinase inhibitors
EZH2	enhancer of zeste homolog 2
EMT	epithelial-mensenchymal transition
ZEB	zinc finger E-box binding homeobox
MMP	matrix metalloproteinase
SIRT1	silent information regulator 2 homolog 1
ATG	autophagy-related gene
